# Meeting Report: Atmospheric Pollution and Human Reproduction

**DOI:** 10.1289/ehp.11074

**Published:** 2008-03-14

**Authors:** Rémy Slama, Lyndsey Darrow, Jennifer Parker, Tracey J. Woodruff, Matthew Strickland, Mark Nieuwenhuijsen, Svetlana Glinianaia, Katherine J. Hoggatt, Srimathi Kannan, Fintan Hurley, Jaroslaw Kalinka, Radim Šrám, Michael Brauer, Michelle Wilhelm, Joachim Heinrich, Beate Ritz

**Affiliations:** 1 Helmholtz Zentrum München, German Research Center for Environmental Health, Institute of Epidemiology, Neuherberg, Germany; 2 INSERM, Avenir Team “Environmental Epidemiology Applied to Fecundity and Reproduction,” Grenoble, France; 3 Université Joseph Fourier Grenoble, Grenoble, France; 4 Rollins School of Public Health, Emory University, Department of Epidemiology, Atlanta, Georgia, USA; 5 National Center for Health Statistics, Office of Analysis and Epidemiology, Hyattsville, Maryland, USA; 6 Program on Reproductive Health and the Environment, University of California, San Francisco, USA; 7 Center for Research in Environmental Epidemiology (CREAL), CIBERESP, IMAM, Barcelona, Spain; 8 Institute of Health and Society, Newcastle University, Newcastle Upon Tyne, United Kingdom; 9 University of Michigan School of Public Health, Ann Arbor, Michigan, USA; 10 Department of Nutrition, University of Massachusetts School of Public Health and Health Sciences, Amherst, Massachusetts, USA; 11 Institute of Occupational Medicine, Edinburgh, United Kingdom; 12 Medical and Environmental Pregnancy Health Hazards Unit, Department of Perinatology, First Chair of Gynecology and Obstetrics, Medical University, Lodz, Poland; 13 Department of Epidemiology, Nofer Institute of Occupational Medicine, Lodz, Poland; 14 Institute of Experimental Medicine, Department of Genetic Ecotoxicology, Prague, Czech Republic; 15 School of Environmental Health, University of British Columbia, Vancouver, British Columbia, Canada; 16 Department of Epidemiology, School of Public Health, University of California, Los Angeles, California

**Keywords:** atmospheric pollution, bias, birth weight, environment, exposure assessment, fecundity, geographic information system, intrauterine growth restriction, particulate matter, pregnancy, reproduction, small for gestational age

## Abstract

**Background:**

There is a growing body of epidemiologic literature reporting associations between atmospheric pollutants and reproductive outcomes, particularly birth weight and gestational duration.

**Objectives:**

The objectives of our international workshop were to discuss the current evidence, to identify the strengths and weaknesses of published epidemiologic studies, and to suggest future directions for research.

**Discussion:**

Participants identified promising exposure assessment tools, including exposure models with fine spatial and temporal resolution that take into account time–activity patterns. More knowledge on factors correlated with exposure to air pollution, such as other environmental pollutants with similar temporal variations, and assessment of nutritional factors possibly influencing birth outcomes would help evaluate importance of residual confounding. Participants proposed a list of points to report in future publications on this topic to facilitate research syntheses. Nested case–control studies analyzed using two-phase statistical techniques and development of cohorts with extensive information on pregnancy behaviors and biological samples are promising study designs. Issues related to the identification of critical exposure windows and potential biological mechanisms through which air pollutants may lead to intrauterine growth restriction and premature birth were reviewed.

**Conclusions:**

To make progress, this research field needs input from toxicology, exposure assessment, and clinical research, especially to aid in the identification and exposure assessment of feto-toxic agents in ambient air, in the development of early markers of adverse reproductive outcomes, and of relevant biological pathways. In particular, additional research using animal models would help better delineate the biological mechanisms underpinning the associations reported in human studies.

After a seminal publication in 1977 (Williams et al. 1977), few studies addressing the possible effects of air pollutants on human reproduction were published before the late 1990s, a time when the number of publications sharply increased. A brief summary of the main findings is given in Table 1. Several reviews exist (Glinianaia et al. 2004a, 2004b; Lacasana et al. 2005; Maisonet et al. 2004; Šrám et al. 2005). Although it is still too early to draw firm conclusions, these data suggest adverse associations between air pollution, specifically carbon monoxide, nitrogen dioxide, sulfur dioxide, and particulate matter [PM; particularly fine particulate matter, PM with aerodynamic diameter < 2.5 μm (PM_2.5_)], and measures of fetal growth (assessed at birth) and gestational duration. For other pollutants (e.g., ozone) and outcomes (e.g., semen quality or birth defects), either the evidence to date is weaker or few data exist.

## Objectives

The International Workshop on Air Pollution and Human Reproduction was convened 9–11 May 2007 to discuss the current body of evidence for effects of atmospheric pollution on human reproduction, to identify the strengths and weaknesses of published epidemiologic studies, to suggest future directions for research, to foster collaboration, and to promote dialogue among epidemiologists, toxicologists, clinicians, and biostatisticians. Several outcomes related to human reproduction were the focus of the discussions, including pregnancy outcomes [intrauterine growth restriction (IUGR), gestational age] and male reproductive health (semen quality). We report here on the issues discussed by the speakers, workshop participants, and working groups; many of these issues and ideas were raised and discussed without any formal process of consensus building and should therefore not be seen as being endorsed by all workshop participants.

## Results

### Study design–related issues

#### Study designs

An approach commonly employed in epidemiologic studies of air pollution and birth outcomes is linkage of outcome and covariate data from birth certificate records with ambient air quality monitoring data. Its main advantage is that it allows conducting large size studies at a very low cost because it relies on routinely collected data. Its limitations are exposure misclassification and possibly confounding. For these reasons, prospective cohort studies with recruitment of women before delivery (e.g., Choi et al. 2006) hold promise: They allow use of biomarkers of exposure or outcome and conduct personal monitoring and collection of detailed information on behaviors related to exposure and on confounders, at a much higher cost. These two designs can be coupled by conducting case–control studies with collection of additional information at the individual level for a sample nested within a cohort constituted from birth records (Ritz et al. 2007); nested studies combine the strength of the larger sample size with more detailed information for a subset of pregnancies.

The time-series approach has proven useful to study the acute cardio-respiratory effects of air pollution and has been adapted to studies of preterm birth and fetal death (Pereira et al. 1998; Sagiv et al. 2005). However, unlike the traditional time-series analysis in which the population at risk (e.g., of cardiac death) remains relatively stable across time, the population at risk of adverse birth outcomes is constantly changing throughout the year. Given that the seasonality of birth has been reported to differ by factors related to socioeconomic status (Bobak and Gjonca 2001), composition of the population at risk may differ across seasons. Thus, application of time-series or case–crossover designs to reproductive outcomes may require additional considerations. Generally, these approaches relying only on temporal variations in exposure appear complementary to the above-mentioned designs relying on cohorts or birth records, which usually take advantage of both spatial and temporal exposure contrasts.

#### Confounding

**Figure 1:**
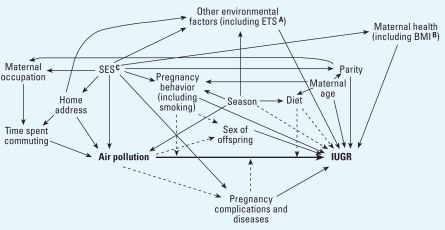
Hypothesized relations between air pollution, IUGR, and extraneous factors possibly acting as confounders in an epidemiologic study of air pollution effects on IUGR. Abbreviations: ETS, environmental tobacco smoke; BMI, body mass index; SES, marker of socioeconomic status (e.g., maternal education). Arrows indicate plausible effects of a factor over another not mediated by another factor present in the diagram. A dotted arrow indicates a plausible although not established relation. An arrow from a factor A that intersects an arrow from B to C indicates that A may modify the effect of B on C (Weinberg 2007).

Because air pollution levels vary in time and space, any factor influencing reproduction and varying with time or space in a way similar to air pollutants is a potential confounder (Figure 1). However, some common pregnancy complications (e.g., preeclampsia) associated with adverse birth outcomes might be caused by air pollutants and should therefore probably not be treated as confounders. The workshop discussions focused on socioeconomic status, season, and nutrition.

Socioeconomic status and related factors are associated with the occurrence of adverse reproductive outcomes (Parker et al. 1994). Part of this association may be explained by variables that we can control for, such as active or passive smoking, parity, body mass index, and occupational and residential exposures to other pollutants. However, some residual influence of socioeconomic status on reproductive outcomes may remain after controlling for these factors. Because socioeconomic status may also be associated with air pollution levels in neighborhoods (Woodruff et al. 2003), it is a potential confounder. Higher levels of primary traffic-related air pollutants are often observed in the city center than in the suburbs; in many U.S. cities, people from poorer socioeconomic classes more often live in the city center than in the suburbs and thus are exposed to higher levels of these pollutants. An opposite pattern may exist in some European cities, where city centers are more often inhabited by residents with higher socioeconomic status. Thus, as exemplified for typical U.S. and European cities, the direction of the implied confounding bias in studies without efficient adjustment for socioeconomic status might depend on the study area. An issue remains about how to measure socioeconomic status to control for it in studies of air pollution and reproduction—for example, about the best way to combine characteristics such as income, educational level or occupation of either partner, ethnicity, type of health insurance; different measures of socioeconomic status probably need to be constructed in each country.

Season is associated with air pollution levels. Moreover, some data suggest that premature births are associated with season (Lee et al. 2006), although part of this association might in fact be attributable to seasonality in air pollutants levels. The underlying cause for an association between premature birth and season might also be exposure to other environmental factors that vary as well with season, such as drinking-water pollutants or infectious diseases; in this case, season should be seen as a potential confounder in studies of air pollution and premature birth. Because season of birth is influenced by the duration of pregnancy, which in turn may be shortened by exposure to air pollutants, and because confounders should, by definition, not be affected by exposure (Rothman and Greenland 1998), the more appropriate adjustment might be for season of conception rather than season of birth. To minimize residual confounding, it may also be necessary to explore smoothing approaches such as spline regression (Salam et al. 2005) rather than employing a simple qualitative approach for coding season. In some settings, the association of season with air pollution might be very strong (particularly with trimester-specific air pollution levels); in this case, controlling for season might produce overadjustment or make the estimates associated with air pollution unstable. Ideally, it would be more appropriate to adjust for the seasonally varying factors underlying any association between season and birth outcome. For similar reasons, season is also a potential confounder in studies of semen quality.

Maternal nutrition before and during pregnancy may vary strongly by geographic area, ethnicity, socioeconomic status, and possibly season and hence with air pollution levels. Animal experiments suggest an influence of maternal nutrition on measures of IUGR (Kind et al. 2006). Currently, very few epidemiologic studies support an effect of variations in maternal diet as currently encountered in industrialized countries on IUGR (Stein et al. 2004); however, in general it would be biologically plausible. There is also recent work showing possible effect measure modification between nutrition and air pollutants. Contrary to season or socioeconomic factors, any confounding by nutritional factors might be difficult to quantify and remove because of measurement errors in the assessment of nutritional factors.

Studying separately the apparent effects of the temporal and spatial components of exposure might also constitute an option for examining potential residual confounding (Janes et al. 2007). The use of a control exposure window after pregnancy might be another way to examine potential for residual confounding by factors spatially correlated with exposure.

#### Effect measure modification

In theory, all potential confounders are candidates for effect measure modification (VanderWeele and Robins 2007). So far, one study estimated stronger effects of air pollution in neighborhoods with low socioeconomic status in winter (Ponce et al. 2005), suggesting an increased vulnerability in these populations. A stronger effect of air pollution on birth weight was also reported for parous than for nulliparous women in a study in which exposure was estimated for the home address; the authors interpreted this heterogeneity in effects as a consequence of the home address–based exposure estimate being more accurate for parous pregnant women because they are more likely to stay at home to take care of their other children than nulliparous women (Ritz and Yu 1999). A study using biomarkers of exposure to air pollutants and passive smoking reported a stronger association between air pollutants and IUGR among women exposed to passive smoking (Perera et al. 2005) than among women not exposed to passive smoking. It has also been suggested that the sizes of air pollutant effect measure differ for male and female offspring (Ghosh et al. 2007). Concerning nutrition, a review (Kannan et al. 2006) and a recent study (Jedrychowski et al. 2007) hypothesized that maternal prepregnancy and gestational nutrition may modulate the harmful effects of prenatal exposures to PM_2.5_ on birth outcomes. Experiments based on transcriptome analysis indicate that several groups of genes involved in immunity and metabolism of xenobiotics are repressed in the placentas of rats with diet-induced IUGR (Buffat et al. 2007). This suggests that the mechanisms of resistance to xenobiotics such as air pollutants may be altered in the case of IUGR induced by a poor diet, and gives some support to a stronger sensitivity to air pollutants for fetuses exposed to other environmental stressors.

Gene–environment interactions with functional genetic polymorphisms implied in the possible biological pathways of action of air pollutants are also worth considering; Wang et al. (2000) highlighted different size effects for maternal occupational exposure to benzene on gestational duration depending on polymorphisms in genes coding for enzymes involved in phase I and phase II metabolism of xenobiotics (*CYP1A1* and *GSTT1*).

### Exposure assessment

#### Pollutants considered

Most studies have focused on routinely measured “criteria” pollutants for which data are more easily available [i.e., CO, NO_2_, O_3_, PM_2.5_, and PM_10_ (PM with aerodynamic diameter < 10 μm)]. Future studies may want to address specific pollutant sources such as road traffic (distinguishing truck and diesel traffic from the other types of vehicles) or pollutants with specific hypotheses regarding biological mechanisms such as ultrafine particulate matter (< 0.1 μm in aerodynamic diameter, either mass or particle number concentration) or polycyclic aromatic hydrocarbons (PAHs). They may also consider expanding their scope to include the evaluation of mixtures of pollutants and possibly determine the composition of PM because the composition, source, and toxicity of equal-size PM can vary according to time and location (Hopke et al. 2006). This may help explain similarities or differences in results for the same criteria pollutant type reported for different regions.

Finally, although most studies have focused on average exposures, considering the effect of peaks in exposure might provide additional insights.

#### Traditional approaches

Air pollution measurements from existing networks of ambient monitoring stations are often used to assess exposure to air pollution within a given distance from a station (typically, studies have used limits from < 1.7 km up to 8 km) or within a given administrative unit (e.g., county). Such approaches allow including large numbers of births. However, they are hindered by exposure misclassification due to unmeasured time–activity patterns, time spent indoors, and local heterogeneity for certain pollutants. Furthermore, a fairly large proportion of women (20–30%) may move during pregnancy (Canfield et al. 2006), making exposure assessment based only on delivery residence problematic.

In principle, simulation studies could be conducted to estimate the extent of exposure variability and contribution of various sources to the total exposure to optimize the exposure assessment [see, e.g., Whitaker et al. (2003) for an example from another field]. Because one cannot *a priori* predict the effect of exposure measurement error (Jurek et al. 2005), sensitivity analyses (Lash and Fink 2003; Zeger et al. 2000) with detailed information concerning the direction and degree of exposure misclassification (e.g., from studies in which several approaches are simultaneously used to assess exposure) would allow quantifying the bias induced by the different sources of measurement error in each study.

#### GIS (geographic information system)–based approaches

Several approaches allow taking into account small area variations in pollution (e.g., presence of a road). Indices such as distance from the closest road or distance-weighted traffic density (Wilhelm and Ritz 2003) constitute a simple source model potentially available in many locales. Exposure estimates can also be derived with land-use regression (LUR) methods, air dispersion models (Brauer et al. 2003; Nieuwenhuijsen et al. 2006), or two-stage geostatistical approaches incorporating monitoring station data and information on temporally or spatially varying covariates (Fanshawe et al. 2007). The resulting increase in spatial resolution of exposure models should not be achieved at the cost of a poorer temporal resolution. Indeed, the critical exposure window for many reproductive outcomes may be short (days, months, or trimesters) and LUR models typically yield yearly exposure estimates. One option is to incorporate temporal variability into LUR models based on measures from background monitoring stations (Brauer et al. 2008; Slama et al. 2007). However, further studies may be needed to determine how well background stations reflect temporal variability at traffic locations.

#### Considering each microenvironment

Because women may spend a considerable amount of their time outside their residence, exposure estimates need to be derived for other locations, such as at work and in transport, to create an integrated personal exposure estimate. The transport environment may make a significant contribution to total exposure, even when the time spent in this environment is short (Kaur et al. 2007; Zhu et al. 2007). Time microenvironment activity diaries have been used to capture people’s movement; global positioning systems also offer possibilities (Nethery et al. 2007).

#### Personal dosimetry

When a sufficient number of measurements are taken (e.g., during the course of pregnancy), personal monitoring (e.g., Choi et al. 2006; Jedrychowski et al. 2007) may provide an estimate of exposure less prone to misclassification than ecologic or semi-individual approaches; implementation costs for the latter, however, are an order of magnitude smaller per individual. Simulation studies that address power (Armstrong 1987) and bias considerations might help determine if the financial resources in a given study are best invested into increasing sample size or improving accuracy of exposure assessment.

#### Biomarkers of exposure

The use of biomarkers of exposure for outdoor air pollutants is currently limited. Some applications include measurement of adducts between PAHs and DNA in maternal or cord blood (Perera et al. 2005), urinary metabolites of benzene, pulmonary markers of combustion of fossil fuels (Kulkarni et al. 2006), and assessment of cotinine, a metabolite of nicotine, in blood or urine. Compared with studies of respiratory morbidity, studies of human reproduction involve special considerations because of physiologic filters (lung epithelium, placental barrier) between the environment and the target organs (e.g., the placenta, gonads, hypothalamo–pituitary axis). Environmental levels may poorly approximate the dose absorbed by these target organs; for example, correlations of 0.5 to 0.7 between personal exposure to PAH present in PM_2.5_ and PAH–DNA adducts in white blood cells have been reported among women (Binková et al. 1996); more moderate correlations (in the 0.2–0.3 range) have been reported in white blood cells PAH–DNA adducts between maternal blood collected within 1 day postpartum and umbilical cord blood collected at delivery (e.g., Perera et al. 2004). Consequently, correlations between atmospheric PAH levels and PAH–DNA levels in cord blood might be weak. Further work is probably warranted to identify and validate biomarkers specific of traffic-related air pollutants.

A limitation is that metabolites of pollutants usually have short half-lives in the body. Thus, researchers employing such biomarkers need to target the relevant exposure window, or perform repeated measurements, unless validation studies show little intraindividual variations in the concentration of the biomarkers considered. In this regard, the assay of adducts between pollutant metabolites and either DNA or proteins (Castano-Vinyals et al. 2004) constitutes an interesting option, as the half-life of these DNA or protein adducts might be longer than that of unbound metabolites.

### Critical exposure windows

Because of typically strong seasonal variations in air pollution levels, there are opportunities to study whether specific periods of pregnancy and of spermatogenesis are more sensitive to air pollutants than others. However, teasing out the critical windows of exposure is challenging because *a*) different pollutants may act during different periods of pregnancy, *b*) routinely measured (and thus evaluated) pollutants may only be proxy markers of the pollutant(s) affecting health, and *c*) pollutant mixtures differ across locations and time. Windows of highest sensitivity reported in studies on air pollution and IUGR that assessed all trimesters or months of pregnancy are presented in Supplemental Material, Figure 1 (online at http://www.ehponline.org/members/2008/11074/suppl.pdf). For each pollutant, there have been very few studies with similar methodologies (e.g., as far as mutual adjustment for other time windows is concerned), which limits between-studies comparisons. Most studies on IUGR used trimester-specific exposure windows. Yet when there are no strong *a priori* biologic hypotheses, investigating finer time scales (e.g., months) might be a more informative and appropriate approach.

#### Identification of critical exposure windows from biological knowledge

Animal experiments (Rocha E Silva et al. 2008) and biological knowledge should guide the definition of exposure windows. In the case of cardiac malformations, for example, one may focus specifically on exposure that occurred no later than in the second month of pregnancy, which corresponds to a period of rapid fetal heart formation. Because current biological knowledge is more limited for other reproductive outcomes, epidemiologic studies sometimes also use a data-driven approach [relying on models summarized in Supplemental Material, Table 1 (online at http://www.ehponline.org/members/2008/11074/suppl.pdf)] by reporting the effect estimates associated with different exposure windows. We now focus on methodologic issues raised by this approach.

#### Methodologic issues

In studies of preterm delivery relying on binomial regression, a methodologic issue in exposure assessment was pointed out by C. Weinberg at the workshop: The time window is sometimes defined with respect to the date of birth (e.g., a 6-week period before birth). In the case of a birth at 34 gestational weeks, this will correspond to the period from 29 to 34 gestational weeks, whereas for a birth at 41 gestational weeks, this corresponds to the period from weeks 36 to 41, which includes the period from 37 to 41 weeks, when a premature birth cannot occur anymore by definition. Alternatively one could employ a matched case–control design in which exposures are averaged over the same gestational period (e.g., from 29 to 34 gestational weeks) for the cases and the matched controls (Huynh et al. 2006). A survival model is another recommended analytical approach, possibly incorporating time-dependent variables (O’ Neill et al. 2003). Last, one could simply truncate exposure at the gestational cutoff for premature births. Such approaches are also recommended when studying spontaneous abortion or stillbirth.

Other methodologic issues were mentioned. Exposures earlier in pregnancy may be more prone to measurement error than those later in pregnancy, both because maternal residence—often used to assign exposure—is usually known only at birth and because women may spend more time at home later than earlier in pregnancy (Nethery 2007).

Another issue is that correcting gestational age using first trimester ultrasound measurements may lead to underestimating effects of environmental pollutants on birth outcomes, if these effects already manifest early in pregnancy and influence fetal growth at the time of the first ultrasound measurement (Slama et al., in press).

#### Pre- and postevent exposures

Studies could examine pre- and postpregnancy windows of exposure. Prepregnancy exposure to air pollutants might entail genetic or epigenetic effects on the male or female gametes (Somers et al. 2004), which might in turn influence pregnancy outcomes.

Slama et al. (2007) have suggested that comparing the estimated effect of pregnancy exposure with that of postnatal exposure (e.g., the 9 months following birth, if one assumes that the relevant exposure window corresponds to the whole pregnancy) may help in discarding specific biases as the explanation of the association between air pollution and reproductive outcomes. Depending on the correlations between postnatal and pregnancy exposures, associations of postnatal exposure with pregnancy outcome would be expected to be weaker than that of pregnancy exposure, if pregnancy exposure has a causal effect. Although there was no consensus among participants on this issue, the idea might be further explored by simulations.

### Biological mechanisms

#### Alteration of maternal–placental exchanges

**Figure 2:**
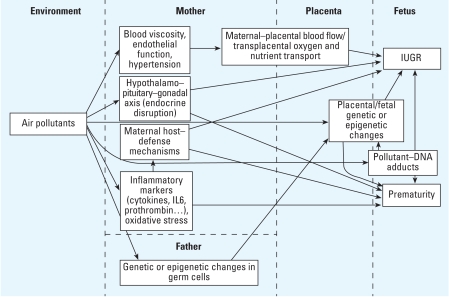
Possible biological mechanisms by which air pollutants could influence IUGR or prematurity. IL, interleukin.

Alterations of utero–placental and umbilical blood flow, and transplacental glucose and oxygen transport influence fetal growth (Pardi et al. 2002). PM levels have been associated with plasma viscosity and endothelial function in nonpregnant adults (Pope and Dockery 2006). Further investigation is necessary to document whether these effects also exist among pregnant women—who differ from other adults in terms of heart rate, plasma viscosity, and insulin resistance (Kaaja and Greer 2005). If so, air pollution-induced changes in plasma viscosity and artery vasoconstriction may in turn influence maternal–placental exchanges and hence fetal growth (Figure 2). This hypothesis could be tested in studies with Doppler measurements of umbilical artery blood flow, which have already been used in studies on maternal exposure to cigarette smoke (Kalinka et al. 2005). Also, some of the studies linking short-term changes in air pollutants to endothelial function or inflammatory response [reviewed, e.g., by Pope and Dockery (2006)] could be repeated among pregnant women.

#### Endocrine disruption

Air pollutants such as heavy metals (cadmium) or diesel exhaust as a whole may interfere with steroidogenesis, may affect progesterone production (Takeda et al. 2004; Tomei et al. 2007), and may thus act as endocrine disrupters. Among pregnant women, endocrine disruption might be involved in causing IUGR (Kanaka-Gantenbein et al. 2003). Endocrine disruption is also a potentially relevant mechanism for effects on male fecundity; male exposures in adulthood, but also during fetal life, should be considered (Sharpe and Irvine 2004).

#### Oxidative pathways and alteration of maternal host–defense mechanisms

PM can induce a broad polyclonal expression of cytokines and chemokines in respiratory epithelium (Sioutas et al. 2005), but also maybe at extrapulmonary sites. Engel et al. (2005) reported that common genetic variants in proinflammatory cytokine genes were associated with spontaneous preterm birth. Future work could study if the effect of PM on preterm birth is modified by polymorphisms in proinflammatory cytokine genes. Oxidative stress pathways are also possibly relevant for male reproductive outcomes, because reactive oxygen species levels have been found to be negatively correlated with sperm motility and concentration (Agarwal et al. 2006). Finally, PM-induced inflammatory processes may modulate host defenses and alter maternal immunity, thus leading to increased susceptibility to infections. These infections may in turn induce preterm labor or IUGR (Figure 2).

#### Paternally mediated effects on birth outcomes

Paternal influences should be considered because of the possible influence of air pollution on semen quality and on heritable mutation rates of male origin (Somers et al. 2004). These male effects might in turn influence reproductive outcomes, although the evidence is currently limited. Attempts to examine in human the influence of air pollution on heritable mutation rates, such as done by Somers et al. (2004) in mice, are worth considering.

#### Animal models

Animal experiments, as well as studies of pregnant women with collection of biological samples may help examine the relevance of these mechanisms. Experimental studies reported alterations of reproductive function in rodents in relation to air pollution (Archibong et al. 2002; Mohallem et al. 2005; Rocha E Silva et al. 2008). The relevance of such results for human reproduction is difficult to discern and human placentation and fetal development (Carter 2007). The guinea pig is a good model for studying placental transfer and fetal growth restriction and the sheep is a well established model for fetal physiology but of limited value for placental research (Carter 2007). The best animal models are nonhuman primates even though their placentation is somewhat different because of their paucity of interstitial trophoblast cells.

### Public health implications

Pregnant women often want to know what they can do to increase the likelihood of the delivery of a healthy child [see Centre for Health and Environment Research (2007) for examples of recommendations given to pregnant women]. Air pollution is also a societal concern. Exposure to ambient air pollution is ubiquitous, and even if increased risks of adverse reproductive outcomes due to such exposures are relatively small, they can have a big impact measured in terms of attributable cases at the population level. One cost–benefit analysis estimated that 200 cases of postneonatal mortality and 10,000 low-birth-weight deliveries would be prevented in the United States between 1990 and 2010 solely through the reduction in air pollutant concentrations expected to occur because of the U.S. Clean Air Act (Wong et al. 2004).

#### Possible effects of air pollutants to consider

Adverse reproductive outcomes might have long-term consequences. IUGR and prematurity have both been linked to increased risk of neonatal mortality, to childhood diseases, and to adult diseases such as heart diseases and diabetes (Barker 2004). The associations between IUGR and neonatal mortality (Wilcox 2001) and between IUGR and health in adulthood (Sinclair et al. 2007) may not be attributable to a causal effect of IUGR per se but rather to some of the determinants of IUGR. Therefore, the long-term health consequences of air pollution–mediated adverse birth outcomes can probably not be well predicted from the known associations between birth weight and health in adulthood, and thus need to be directly assessed. The situation might differ for the long-term consequences of air pollution-mediated premature births.

## Conclusion

Research exploring the effects of air pollution on human reproduction is a young field. Many of the current methodologic issues are shared with other research areas focused on health effects of air pollutants. We indicate here some of its specificities.

Air pollution levels and probably fetal sensitivity to environmental pollutants vary sharply over time, so exposure models should aim toward a fine temporal resolution (this also applies to other reproductive outcomes such as menstrual cycle function). Pregnancy is a period of life with specific time–activity, work, and residential mobility patterns, which must be taken into account. Not only maternal but also paternal exposures are possibly important. In addition to spatial confounding (i.e., by factors spatially correlated with exposure), which may also exist in other environmental studies, reproductive studies can be affected by temporal confounding due to risk factors that vary seasonally with exposure. In terms of identifying biological mechanisms, close collaboration between epidemiology and other basic science disciplines is still missing. The identification of a plausible set of biological mechanisms by biologists, toxicologists, and epidemiologists would give more weight to the associations reported in human observational studies. Given the heterogeneous chemical and physical nature of pollutants such as PM, there is no reason to believe in the existence of a unique biological mechanism likely to explain PM effects on complex events such as fetal growth and premature birth.

## Recommendations

- In addition to the already broadly targeted reproductive outcomes discussed above, other perinatal end points may be sensitive to air pollutant exposures and could be considered in future studies to broaden the case for reproductive outcomes (Table 2).
- We suggested points to report (possibly in online supplements of journals) in the interest of facilitating comparisons across studies in future epidemiologic studies on air pollution and human reproduction (Table 3).
- The spatial resolution of exposure models is often inadequate and is in need of improvement (e.g., by using dispersion and LUR models); these models should also include a temporal component. Time–activity patterns of subjects should be taken into account.
- The development of biomarkers of exposure to traffic-related air pollutants should be encouraged—specifically biomarkers reflecting the dose absorbed by relevant target organs such as the feto-placental unit. This would allow quantification of how the feto-placental dose relates to maternal dose, to environmental levels of pollutants and to the occurrence of adverse reproductive outcomes.
- Investigating the short-term effects of air pollution on endothelial function, inflammatory response, and blood pressure of pregnant women could help understanding if these are possible pathways for air pollutants effects on reproductive outcomes.
- Animal experiments are needed to help identify relevant biological mechanisms.
- The research field has developed through studies on a large number of births making use of existing air quality monitoring and electronic birth certificate data; the utility of this design has been recognized, but it should not be considered the only option. Studies that collect detailed exposure and covariate information and biological samples, possibly in nested subgroups of larger populations, should be further encouraged.
- Study designs that have proven useful in assessing air pollution impacts on other health outcomes (e.g., time-series, case–crossover designs) could be further explored in the context of reproductive outcomes.

## Figures and Tables

**Table 1:** Overview of current evidence concerning the possible effects of air pollutants on human reproduction.

Reproductive health outcome (strength of evidence)	Exposure assessment	Study design	Illustrative references
Male reproductive health			
Semen quality (−/+)	AQMS, biomarkers	Longitudinal or cross-sectional	Rosa et al. 2003; Rubes et al. 2005
Female reproductive health			
Hormonal function (LD)		Experimental (rats)	Archibong et al. 2002
Couples’ fecundity (LD)	AQMS	Pregnancy-based retrospective study; experimental (mice)	Dejmek et al. 2000; Mohallem et al. 2005
Pregnancy and fetal health			
Stillbirth (LD)	AQMS	Time series	Pereira et al. 1998
Prematurity (−/+)	AQMS	Birth register–based study; time series	Huynh et al. 2006; Sagiv et al. 2005; Wilhelm and Ritz 2003
Congenital malformations (−/+)	AQMS	Birth defect register–based study	Ritz et al. 2002
Intrauterine growth, birth weight (+)	AQMS, biomarkers, LUR, personal monitoring	Birth register–based study; cohorts of pregnant women; experimental	Choi et al. 2006; Ritz and Yu 1999; Rocha E Silva et al. 2008; Slama et al. 2007
Secondary sex-ratio (LD)	AQMS	Birth register–based study and experiment (mice)	Lichtenfels et al. 2007
Postnatal health			
Infant death (+)	AQMS	Case–control study relying on birth/death certificates.	Ritz et al. 2006; Woodruff et al. 2006
Transgenerational effects			
Heritable mutation rate (LD)	Personal monitoring	Experimental (mice)	Somers et al. 2004

Abbreviations: +, suggestive evidence; −/+, mixed or yet inconclusive results; AQMS, air quality monitoring stations; LD, limited data, indicates outcomes little or not studied; LUR, land-use regression models.

**Table 2:** Suggested reproductive outcomes to study in relation to atmospheric pollutants.

Prepregnancy events	Pregnancy events	Postpregnancy events
Time to pregnancy^a^	Spontaneous abortions, stillbirths	Placental size, weight
Semen quality^a^	Maternal hypertension, pulse pressure	Testicle, penis sizes
Menstrual cycle^a^	Preeclampsia	Ano-genital distance (males)
Proteomic markers of sperm function^b^	Fetal ultrasound measurements	Kidney size (boys and girls)
	Fetal growth velocity	Dubowitz or Ballard scores
	Birth weight (*z*-score)	
	Transcriptomic analysis^c^	
	Symmetric vs. asymmetric growth restriction	
	Doppler umbilical artery velocimetry	
	Birth defects	
	Sex ratio	

a Both exposure in adulthood and during intrauterine life are worth considering (e.g., Jensen et al. 2004).

b Lefievre et al. (2007).

c See, for example, Buffat et al. (2007).

**Table 3:** Recommended points to report in epidemiologic studies of the effects of air pollutants on human reproduction.

Topic	Points to report^a^
Population	Characteristics of excluded subjects [see, e.g., Table 1 in Parker et al. (2005)]
Health outcome	Indicate all health outcomes examined
	Birth weight for gestation standards used for SGA classification
	Methods for determining gestational age
Exposure	Rationale behind monitoring station buffer area size, if applicable
	Type of monitoring stations used (e.g., background, source oriented sites)
	Distribution of exposure during the considered time-windows
	Correlation between (window-specific) exposure variables [see, e.g., Table 5 in Parker et al. (2005)]
	Information used to geocode addresses (e.g., ZIP code only vs. street address)
Other covariates	Which socioeconomic factors (or their proxies) were tested, and how do they relate to the exposure and outcome?
Statistical analysis	Check for nonlinear relations between exposure and outcome
	Indicate which adjustment factors had the greatest influence on the estimated effect of exposure

SGA, small for gestational age.

a For more general recommendations on points to report see, for example, von Elm et al. (2007).

## References

[b1-ehp0116-000791] Agarwal A, Sharma RK, Nallella KP, Thomas AJ, Alvarez JG, Sikka SC (2006). Reactive oxygen species as an independent marker of male factor infertility. Fertil Steril.

[b2-ehp0116-000791] Archibong AE, Inyang F, Ramesh A, Greenwood M, Nayyar T, Kopsombut P (2002). Alteration of pregnancy related hormones and fetal survival in F-344 rats exposed by inhalation to benzo(*a*)pyrene. Reprod Toxicol.

[b3-ehp0116-000791] Armstrong B (1987). A simple estimator of minimum detectable relative risk, sample size, or power in cohort studies. Am J Epidemiol.

[b4-ehp0116-000791] Barker DJ (2004). The developmental origins of adult disease. J Am Coll Nutr.

[b5-ehp0116-000791] Binková B, Lewtas J, Mísková I, Rössner P, Cerná M, Mrácková G (1996). Biomarker studies in northern Bohemia. Environ Health Perspect.

[b6-ehp0116-000791] Bobak M, Gjonca A (2001). The seasonality of live birth is strongly influenced by socio-demographic factors. Hum Reprod.

[b7-ehp0116-000791] Brauer M, Hoek G, van Vliet P, Meliefste K, Fischer P, Gehring U (2003). Estimating long-term average particulate air pollution concentrations: application of traffic indicators and geographic information systems. Epidemiology.

[b8-ehp0116-000791] Brauer M, Lencar C, Tamburic L, Koehoorn M, Demers P, Karr C (2008). Cohort study of traffic-related air pollution Impacts on birth outcomes. Environ Health Perspect.

[b9-ehp0116-000791] Buffat C, Mondon F, Rigourd V, Boubred F, Bessieres B, Fayol L (2007). A hierarchical analysis of transcriptome alterations in intrauterine growth restriction (IUGR) reveals common pathophysiological pathways in mammals. J Pathol.

[b10-ehp0116-000791] Canfield MA, Ramadhani TA, Langlois PH, Waller DK (2006). Residential mobility patterns and exposure misclassification in epidemiologic studies of birth defects. J Expo Sci Environ Epidemiol.

[b11-ehp0116-000791] Carter AM (2007). Animal models of human placentation—a review. Placenta.

[b12-ehp0116-000791] Castano-Vinyals G, D’Errico A, Malats N, Kogevinas M (2004). Biomarkers of exposure to polycyclic aromatic hydrocarbons from environmental air pollution. Occup Environ Med.

[b13-ehp0116-000791] Centre for Health and Environment Research (2007). Pregnancy, Health and Air Pollution.

[b14-ehp0116-000791] Choi H, Jedrychowski W, Spengler J, Camann DE, Whyatt RM, Rauh V (2006). International studies of prenatal exposure to polycyclic aromatic hydrocarbons and fetal growth. Environ Health Perspect.

[b15-ehp0116-000791] Dejmek J, Jelinek R, Solansky I, Beneš I, Šrám RJ (2000). Fecundability and parental exposure to ambient sulfur dioxide. Environ Health Perspect.

[b16-ehp0116-000791] Engel SA, Erichsen HC, Savitz DA, Thorp J, Chanock SJ, Olshan AF (2005). Risk of spontaneous preterm birth is associated with common proinflammatory cytokine polymorphisms. Epidemiology.

[b17-ehp0116-000791] Fanshawe TR, Diggle PJ, Rushton S, Sanderson R, Lurz PWW, Glinianaia SV (2007). Modelling spatio-temporal variation in exposure to particulate matter: a two-stage approach. Environmetrics.

[b18-ehp0116-000791] Ghosh R, Rankin J, Pless-Mulloli T, Glinianaia S (2007). Does the effect of air pollution on pregnancy outcomes differ by gender? A systematic review. Environ Res.

[b19-ehp0116-000791] Glinianaia SV, Rankin J, Bell R, Pless-Mulloli T, Howel D (2004a). Does particulate air pollution contribute to infant death? A systematic review. Environ Health Perspect.

[b20-ehp0116-000791] Glinianaia SV, Rankin J, Bell R, Pless-Mulloli T, Howel D (2004b). Particulate air pollution and fetal health: a systematic review of the epidemiologic evidence. Epidemiology.

[b21-ehp0116-000791] Hopke PK, Ito K, Mar T, Christensen WF, Eatough DJ, Henry RC (2006). PM source apportionment and health effects: 1. Intercomparison of source apportionment results. J Expo Sci Environ Epidemiol.

[b22-ehp0116-000791] Huynh M, Woodruff TJ, Parker JD, Schoendorf KC (2006). Relationships between air pollution and preterm birth in California. Paediatr Perinat Epidemiol.

[b23-ehp0116-000791] Janes H, Dominici F, Zeger SL (2007). Trends in air pollution and mortality: an approach to the assessment of unmeasured confounding. Epidemiology.

[b24-ehp0116-000791] Jedrychowski W, Masters E, Choi H, Sochacka E, Flak E, Mroz E (2007). Pre-pregnancy dietary vitamin A intake may alleviate the adverse birth outcomes associated with pre-natal pollutant exposure: epidemiologic cohort study in Poland. Int J Occup Environ Health.

[b25-ehp0116-000791] Jensen TK, Jorgensen N, Punab M, Haugen TB, Suominen J, Zilaitiene B (2004). Association of *in utero* exposure to maternal smoking with reduced semen quality and testis size in adulthood: a cross-sectional study of 1,770 young men from the general population in five European countries. Am J Epidemiol.

[b26-ehp0116-000791] Jurek AM, Greenland S, Maldonado G, Church TR (2005). Proper interpretation of non-differential misclassification effects: expectations vs observations. Int J Epidemiol.

[b27-ehp0116-000791] Kaaja RJ, Greer IA (2005). Manifestations of chronic disease during pregnancy. JAMA.

[b28-ehp0116-000791] Kalinka J, Hanke W, Sobala W (2005). Impact of prenatal tobacco smoke exposure, as measured by midgestation serum cotinine levels, on fetal biometry and umbilical flow velocity waveforms. Am J Perinatol.

[b29-ehp0116-000791] Kanaka-Gantenbein C, Mastorakos G, Chrousos GP (2003). Endocrine-related causes and consequences of intrauterine growth retardation. Ann NY Acad Sci.

[b30-ehp0116-000791] Kannan S, Misra DP, Dvonch JT, Krishnakumar A (2006). Exposures to airborne particulate matter and adverse perinatal outcomes: a biologically plausible mechanistic framework for exploring potential effect modification by nutrition. Environ Health Perspect.

[b31-ehp0116-000791] Kaur S, Nieuwenhuijsen M, Colvile RN (2007). Fine particulate matter and carbon monoxide exposure concentrations in urban street transport microenvironments. Atmos Environ.

[b32-ehp0116-000791] Kind KL, Moore VM, Davies MJ (2006). Diet around conception and during pregnancy—effects on fetal and neonatal outcomes. Reprod Biomed Online.

[b33-ehp0116-000791] Kulkarni N, Pierse N, Rushton L, Grigg J (2006). Carbon in airway macrophages and lung function in children. N Engl J Med.

[b34-ehp0116-000791] Lacasana M, Esplugues A, Ballester F (2005). Exposure to ambient air pollution and prenatal and early childhood health effects. Eur J Epidemiol.

[b35-ehp0116-000791] Lash TL, Fink AK (2003). Semi-automated sensitivity analysis to assess systematic errors in observational data. Epidemiology.

[b36-ehp0116-000791] Lee SJ, Steer PJ, Filippi V (2006). Seasonal patterns and preterm birth: a systematic review of the literature and an analysis in a London-based cohort. BJOG.

[b37-ehp0116-000791] Lefievre L, Bedu-Addo K, Conner SJ, Machado-Oliveira GS, Chen Y, Kirkman-Brown JC (2007). Counting sperm does not add up any more: time for a new equation?. Reproduction.

[b38-ehp0116-000791] Lichtenfels AJ, Gomes JB, Pieri PC, El Khouri Miraglia SG, Hallak J, Saldiva PH (2007). Increased levels of air pollution and a decrease in the human and mouse male-to-female ratio in São Paulo, Brazil. Fertil Steril.

[b39-ehp0116-000791] Maisonet M, Correa A, Misra D, Jaakkola JJ (2004). A review of the literature on the effects of ambient air pollution on fetal growth. Environ Res.

[b40-ehp0116-000791] Mohallem SV, de Araujo Lobo DJ, Pesquero CR, Assuncao JV, de Andre PA, Saldiva PH (2005). Decreased fertility in mice exposed to environmental air pollution in the city of São Paulo. Environ Res.

[b41-ehp0116-000791] Nethery E (2007). From Measures to Models: Predicting Exposure to Air Pollution among Pregnant Women [MSc Thesis].

[b42-ehp0116-000791] Nethery E, Leckie SE, Teschke K, Brauer M (2007). From measures to models: An evaluation of air pollution exposure assessment for epidemiologic studies of pregnant women. Occup Environ Med.

[b43-ehp0116-000791] Nieuwenhuijsen M, Paustenbach D, Duarte-Davidson R (2006). New developments in exposure assessment: the impact on the practice of health risk assessment and epidemiological studies. Environ Int.

[b44-ehp0116-000791] O’Neill M, Hertz-Picciotto I, Pastore LM, Weatherley BD (2003). Have studies of urinary tract infection and preterm delivery used the most appropriate methods?. Paediatr Perinat Epidemiol.

[b45-ehp0116-000791] Pardi G, Marconi AM, Cetin I (2002). Placental-fetal interrelationship in IUGR fetuses—a review. Placenta.

[b46-ehp0116-000791] Parker JD, Schoendorf KC, Kiely JL (1994). Associations between measures of socioeconomic status and low birth weight, small for gestational age, and premature delivery in the United States. Ann Epidemiol.

[b47-ehp0116-000791] Parker JD, Woodruff TJ, Basu R, Schoendorf KC (2005). Air pollution and birth weight among term infants in California. Pediatrics.

[b48-ehp0116-000791] Pereira LA, Loomis D, Conceicao GM, Braga AL, Arcas RM, Kishi HS (1998). Association between air pollution and intrauterine mortality in São Paulo, Brazil. Environ Health Perspect.

[b49-ehp0116-000791] Perera FP, Tang D, Rauh V, Lester K, Tsai WY, Tu YH (2005). Relationships among polycyclic aromatic hydrocarbon-DNA adducts, proximity to the World Trade Center, and effects on fetal growth. Environ Health Perspect.

[b50-ehp0116-000791] Perera FP, Tang D, Tu YH, Cruz LA, Borjas M, Bernert T (2004). Biomarkers in maternal and newborn blood indicate heightened fetal susceptibility to procarcinogenic DNA damage. Environ Health Perspect.

[b51-ehp0116-000791] Ponce NA, Hoggatt KJ, Wilhelm M, Ritz B (2005). Preterm birth: the interaction of traffic-related air pollution with economic hardship in Los Angeles neighborhoods. Am J Epidemiol.

[b52-ehp0116-000791] Pope CA, Dockery DW (2006). Health effects of fine particulate air pollution: lines that connect. J Air Waste Manag Assoc.

[b53-ehp0116-000791] Ritz B, Wilhelm M, Hoggatt KJ, Ghosh JK (2007). Ambient air pollution and preterm birth in the environment and pregnancy outcomes study at the University of California, Los Angeles. Am J Epidemiol.

[b54-ehp0116-000791] Ritz B, Wilhelm M, Zhao Y (2006). Air pollution and infant death in southern California, 1989–2000. Pediatrics.

[b55-ehp0116-000791] Ritz B, Yu F (1999). The effect of ambient carbon monoxide on low birth weight among children born in southern California between 1989 and 1993. Environ Health Perspect.

[b56-ehp0116-000791] Ritz B, Yu F, Fruin S, Chapa G, Shaw GM, Harris JA (2002). Ambient air pollution and risk of birth defects in southern California. Am J Epidemiol.

[b57-ehp0116-000791] Rocha E, Silva IR, Lichtenfels AJ, Amador Pereira LA, Saldiva PH (2008). Effects of ambient levels of air pollution generated by traffic on birth and placental weights in mice. Fertil Steril.

[b58-ehp0116-000791] Rosa MD, Zarrilli S, Paesano L, Carbone U, Boggia B, Petretta M (2003). Traffic pollutants affect fertility in men. Hum Reprod.

[b59-ehp0116-000791] Rothman KJ, Greenland S (1998). Modern Epidemiology.

[b60-ehp0116-000791] Rubes J, Selevan SG, Evenson DP, Zudova D, Vozdova M, Zudova Z (2005). Episodic air pollution is associated with increased DNA fragmentation in human sperm without other changes in semen quality. Hum Reprod.

[b61-ehp0116-000791] Sagiv SK, Mendola P, Loomis D, Herring AH, Neas LM, Savitz DA (2005). A time-series analysis of air pollution and preterm birth in Pennsylvania, 1997–2001. Environ Health Perspect.

[b62-ehp0116-000791] Salam MT, Millstein J, Li YF, Lurmann FW, Margolis HG, Gilliland FD (2005). Birth outcomes and prenatal exposure to ozone, carbon monoxide, and particulate matter: results from the Children’s Health Study. Environ Health Perspect.

[b63-ehp0116-000791] Sharpe RM, Irvine DS (2004). How strong is the evidence of a link between environmental chemicals and adverse effects on human reproductive health?. BMJ.

[b64-ehp0116-000791] Sinclair SK, Lea RG, Rees WD, Young LE (2007). The developmental origins of health and disease: current theories and epigenetic mechanisms. Soc Reprod Fertil Suppl.

[b65-ehp0116-000791] Sioutas C, Delfino R, Singh M (2005). Exposure assessment for atmospheric ultrafine particles (UFPs) and implications for epidemiologic research. Environ Health Perspect.

[b66-ehp0116-000791] Slama R, Khoshnood B, Kaminski M How to control for gestational age in studies of effects of environmental factors on fetal growth? [Letter]. Environ Health Perspect.

[b67-ehp0116-000791] Slama R, Morgenstern V, Cyrys J, Zutavern A, Herbarth O, Wichmann HE (2007). Traffic-related atmospheric pollutant levels during pregnancy and offspring’s term birth weight: a study relying on a land-use regression exposure model. Environ Health Perspect.

[b68-ehp0116-000791] Somers CM, McCarry BE, Malek F, Quinn JS (2004). Reduction of particulate air pollution lowers the risk of heritable mutations in mice. Science.

[b69-ehp0116-000791] Šrám RJ, Binková B, Dejmek J, Bobak M (2005). Ambient air pollution and pregnancy outcomes: a review of the literature. Environ Health Perspect.

[b70-ehp0116-000791] Stein AD, Zybert PA, van de Bor M, Lumey LH (2004). Intrauterine famine exposure and body proportions at birth: the Dutch Hunger Winter. Int J Epidemiol.

[b71-ehp0116-000791] Takeda K, Tsukue N, Yoshida S (2004). Endocrine-disrupting activity of chemicals in diesel exhaust and diesel exhaust particles. Environ Sci.

[b72-ehp0116-000791] Tomei G, Ciarrocca M, Bernardini A, Capozzella A, Rosati MV, Anzelmo V (2007). Plasma 17-alpha-OH-progesterone in male workers exposed to traffic pollutants. Ind Health.

[b73-ehp0116-000791] VanderWeele TJ, Robins JM (2007). Four types of effect modification: a classification based on directed acyclic graphs. Epidemiology.

[b74-ehp0116-000791] von Elm E, Altman DG, Egger M, Pocock SJ, Gotzsche PC, Vandenbroucke JP (2007). The Strengthening the Reporting of Observational Studies in Epidemiology (STROBE) statement: guidelines for reporting observational studies. Ann Intern Med.

[b75-ehp0116-000791] Wang X, Chen D, Niu T, Wang Z, Wang L, Ryan L (2000). Genetic susceptibility to benzene and shortened gestation: evidence of gene-environment interaction. Am J Epidemiol.

[b76-ehp0116-000791] Weinberg CR (2007). Can DAGs clarify effect modification?. Epidemiology.

[b77-ehp0116-000791] Whitaker HJ, Nieuwenhuijsen MJ, Best NG (2003). The relationship between water concentrations and individual uptake of chloroform: a simulation study. Environ Health Perspect.

[b78-ehp0116-000791] Wilcox AJ (2001). On the importance—and the unimportance—of birthweight. Int J Epidemiol.

[b79-ehp0116-000791] Wilhelm M, Ritz B (2003). Residential proximity to traffic and adverse birth outcomes in Los Angeles County, California, 1994–1996. Environ Health Perspect.

[b80-ehp0116-000791] Williams L, Spence A, Tideman SC (1977). Implications of the observed effects of air pollution on birth weight. Soc Biol.

[b81-ehp0116-000791] Wong EY, Gohlke J, Griffith WC, Farrow S, Faustman EM (2004). Assessing the health benefits of air pollution reduction for children. Environ Health Perspect.

[b82-ehp0116-000791] Woodruff TJ, Parker JD, Kyle AD, Schoendorf KC (2003). Disparities in exposure to air pollution during pregnancy. Environ Health Perspect.

[b83-ehp0116-000791] Woodruff TJ, Parker JD, Schoendorf KC (2006). Fine particulate matter (PM_2.5_) air pollution and selected causes of postneonatal infant mortality in California. Environ Health Perspect.

[b84-ehp0116-000791] Zeger SL, Thomas D, Dominici F, Samet JM, Schwartz J, Dockery D (2000). Exposure measurement error in time-series studies of air pollution: concepts and consequences. Environ Health Perspect.

[b85-ehp0116-000791] Zhu Y, Eiguren-Fernandez A, Hinds WC, Miguel AH (2007). In-cabin commuter exposure to ultrafine particles on Los Angeles freeways. Environ Sci Technol.

